# Supplementary figures and images for: The effect of prior long-term recellularization with keratocytes of decellularized porcine corneas implanted in a rabbit anterior lamellar keratoplasty model
Source: PLoS One. 2021 Jun 1;16(6):e0245406. doi: 10.1371/journal.pone.0245406 (PMC8168847; doi:10.1371/journal.pone.0245406)

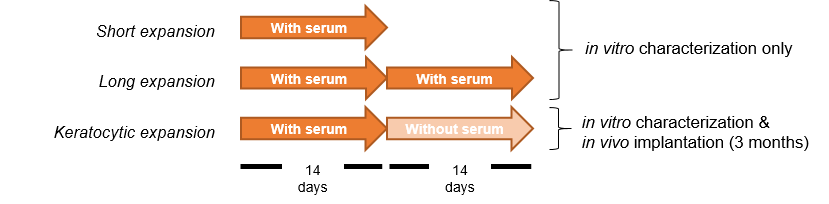

Supplement: S1 Fig — (TIF) [file pone.0245406.s001.tif]
