# Supplementary material for: The effect of prior long-term recellularization with keratocytes of decellularized porcine corneas implanted in a rabbit anterior lamellar keratoplasty model
Source: PLoS One. 2021 Jun 1;16(6):e0245406. doi: 10.1371/journal.pone.0245406 (PMC8168847; doi:10.1371/journal.pone.0245406)
Supplement: S1 Raw image — (PDF) [file pone.0245406.s002.pdf]

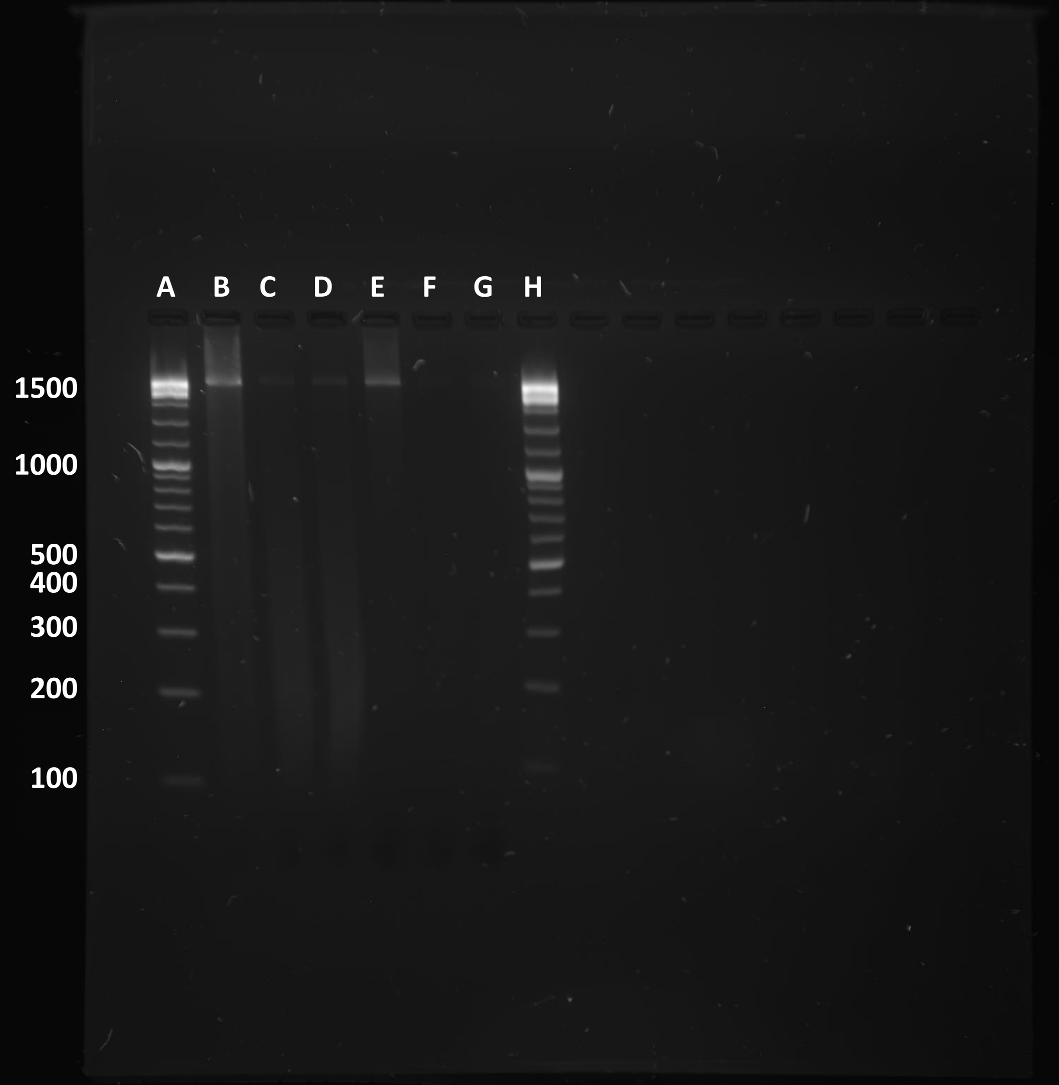

A: Ladder
B: Native cornea
C: Decellularized cornea
D: Decellularized cornea
E: Native cornea
F: Blank
G: Blank
H: Ladder
